# Impact of the COVID-19 pandemic on hospital-based heart failure care in New South Wales, Australia: a linked data cohort study

**DOI:** 10.1186/s12913-024-11840-0

**Published:** 2024-11-08

**Authors:** Daniel McIntyre, Desi Quintans, Samia Kazi, Haeri Min, Wen-Qiang He, Simone Marschner, Rohan Khera, Natasha Nassar, Clara K. Chow

**Affiliations:** 1 Westmead Applied Research Centre, Faculty of Medicine and Health, University of Sydney, Sydney, NSW 2145 Australia; 2 Child Population and Translational Health Research, Children’s Hospital at Westmead Clinical School, Faculty of Medicine and Health, University of Sydney, Sydney, NSW Australia; 3 Menzies Centre for Health Policy and Economics, Faculty of Medicine and Health, University of Sydney, Sydney, NSW Australia; 4 Charles Perkins Centre, University of Sydney, Westmead, NSW Australia; 5 Department of Cardiology, Westmead Hospital, Westmead, Australia; 6 Section of Cardiovascular Medicine, Department of Internal Medicine, Yale School of Medicine, New Haven, CT USA; 7 Section of Biomedical Informatics and Data Science, Yale School of Medicine, New Haven, CT USA; 8 Center for Outcomes Research and Evaluation, Yale-New Haven Hospital, New Haven, CT USA

**Keywords:** COVID-19, Heart failure, Health service utilisation

## Abstract

**Background:**

Healthcare policy implemented during the COVID-19 pandemic may have impacted the health of patients with heart failure. Australian data provide a unique opportunity to examine service disruption independent of significant COVID-19 burden. This study aimed to examine heart failure care during the pandemic in New South Wales (NSW).

**Methods:**

Analysis of hospital utilisation among patients aged ≥ 18 years with a primary diagnosis of heart failure conducted using linked administrative health records from hospital admission, emergency department, non-admitted services, and mortality data collections. Health service utilisation and outcomes were compared “Pre-pandemic” (PP): 16th March 2018 – 28th August 2019 and “During pandemic” (DP): 16th March 2020 – 28th August 2021. Mortality data were available until December 2021.

**Results:**

Heart failure-related ED presentations and hospital admissions were similar between the periods (PP = 15,324 vs DP = 15,023 ED presentations, PP = 24,072 vs DP = 23,145 hospital admissions), though rates of admission from ED were lower DP (PP = 12,783/15,324 (83.4% [95% CI 82.8-84.0]) vs DP = 12,230/15,023 (81.4% [95% CI 80.8-82.0%]). There was no difference according to age, sex, rurality, or socioeconomic status. Outpatient volume reduced DP (PP = 44,447 vs DP = 35,801 occasions of service), but telehealth visits increased nearly threefold (PP = 5,978/44,447 (13.4% [95% CI 13.1-13.8%]) vs DP = 15,901/35,801 (44.4% [95% CI 43.9-44.9%]) with highest uptake among the wealthy and those in major cities. Time to heart failure-related ED presentation, hospitalisation or all-cause mortality following index admission was longer DP (PP = 273 [IQR 259, 290] days, DP = 323 [IQR 300, 342] days, HR 0.91 [95% CI 0.88, 0.95]).

**Conclusions:**

Policies implemented DP had minimal impact on volumes of inpatient heart failure care in NSW hospitals, but there were fewer admissions from ED and reduced volumes of publicly funded outpatient care. A rapid shift from patient-facing to remotely delivered care enabled compliance with restrictions and was associated with increased time to heart failure-related adverse events, but access was not afforded equally across the socio-demographic spectrum.

## Background

The COVID-19 pandemic has been the most significant global health emergency in modern history. Initially considered a respiratory illness, the cardiovascular sequelae of COVID-19 are now well understood [1–3]. However, it is more difficult to quantify how restrictions implemented to reduce disease spread have impacted cardiovascular health. Some have argued that reduced industrial activity with consequential pollution reduction, especially in developing countries, contributed to improved air quality, a potential net positive impact [4]. However, it is also possible that the rapid system wide shifts to telehealth-facilitated care coupled with patient hesitancy to present for fear of contracting COVID-19 resulted in sub-optimal care for the most vulnerable cardiovascular patients. Patients with heart failure are at higher risk of severe COVID-19 infection, more likely to come from disadvantaged socio-cultural backgrounds, [5] and are hence a critical barometer of equity in cardiovascular care during the pandemic.

Heart failure hospital admissions declined during the pandemic, but outcomes among those hospitalised were worse. In an early analysis of 1,372 patients hospitalised with heart failure in a large centre in London, the volume of hospitalisations during lockdown periods was lower, but in-hospital mortality increased (hazard ratio [HR] 2.23 95% confidence interval [95% CI] 1.34-3.72) [6]. Similar findings are reported from single-centre analyses in Canada [7], Spain [8], and the USA [9]. There is also evidence patients with heart failure in the community had poorer outcomes. A study of the UK-based National Heart Failure audit (*n* = 36, 974) found a decrease in in-hospital mortality (incidence rate ratio [IRR] 0.71 [95% CI 0.67 – 0.75], but an increase in deaths from heart failure at home (IRR 1.31 [95% CI 1.24 – 1.39]) [10].

There are few examples of system level analyses of heart failure care within Australia during the pandemic. Available data suggest abrupt declines in emergency department (ED) presentations, greatest among the most disadvantaged patients [11]. A small study in a single centre in Melbourne found lower volumes of heart failure presentations with higher acuity than pre-COVID [12]. Concomitant low case numbers and strict public health restrictions [13] in Australia provide a unique opportunity to study the impacts of disaster-related health system changes independent of cofounding COVID-19 burden to inform healthcare service planning during future disaster periods.

In this study, we present analyses of inpatient, emergency, and non-admitted heart failure care across New South Wales (NSW), the most populous state in Australia, prior to and during the COVID-19 pandemic. We aim to describe presentation trends, acuity, and outcomes of patients seeking heart failure care during the pandemic compared to pre-pandemic years.

## Methods

### Study population and data sources

#### COVID-19 spread in Australia

The COVID-19 spread in 3 waves across Australia in 2020-2021. Wave 1 occurred between March and May 2020, peaking on 7th April 2020 with a high of 7 COVID-19-related deaths per day. Wave 2 was primarily driven by infections in Victoria between July and November 2020 and peaked on 4th September 2020 at 9 deaths per day. Wave 3 occurred between July and September 2021, peaking on 27th October 2021 with 27 daily deaths. As vaccination was introduced, morbidity from infection reduced, with 357/2,252 (16.0%) of active cases hospitalised in wave 1 compared to 5,021/373,226 (1.3%) in wave 4 in 2022 [14]. Detailed timelines of Australian government restrictions are available elsewhere [15], but ranged from restrictions on large gatherings and hospitality venues at the beginning of the pandemic, to state-wide “lockdowns”, during which residents were required to remain within a prescribed radius of their home other than for essential services, including healthcare.

#### Study exposure periods

The study exposure periods were stratified into before- and during-pandemic time periods according to the effective dates of NSW Public Health Orders relating to COVID-19 (archived at https://legislation.nsw.gov.au/information/covid19-legislation). Patients were included in a study exposure period based on the date of presentation to ED, admission to hospital, or outpatient service delivery. The during-pandemic (DP) period was defined from 16th March 2020 until 28th August 2021, and represents the period during which restriction on movement or regular activities was applied to people and businesses in NSW. The pre-pandemic (PP) period was defined as the period 2 calendar years before this (i.e. 16th March 2018 to 28th August 2019), with an approximate 6-month gap between the two periods.

#### Study cohort

The study cohort included patients aged ≥ 18 admitted to a hospital, presenting to an ED or receiving a hospital-based outpatient service in NSW with a primary diagnosis of heart failure.

Patients were excluded if they ever had a heart failure primary diagnosis before 18 years old, missing age or sex at birth data, sex at birth differed between records, recorded as deceased in any dataset but without a death entry in the registry of births deaths and marriages (RBDM), and/or recorded as deceased multiple times with different death dates.

#### Data sources

We used linked data from the NSW Admitted Patient Data Collection (APDC), Emergency Department Data Collection (EDDC), Non-Admitted Patients (NAP) data collection, the Cause of Death Unit Record File (CODURF), and the RBDM death records from March 2018 to December 2021. These datasets contain information on patient demographic characteristics (age, sex, rurality, socio-economic status), diagnoses, length of hospital stay, admission to intensive care units (ICU), ED triage category and length of hospital stay. Diagnoses in these datasets were recorded using the International Classification of Diseases (ICD; either the earlier 9th Edition, ICD-9, or the Australian modification of the 10th Edition, ICD-10-AM) or SNOMED clinical terms. SNOMED codes were mapped to ICD-10-AM codes using CSIRO’s SnoMAP Starter Web Service (https://ontoserver.csiro.au/site/our-solutions/snomap-starter/). Data linkage was performed probabilistically by the NSW Centre for Health Record Linkage (CHeReL) with an estimated false positive rate of 0.5%.

Heart failure was identified using relevant diagnosis fields in each data collection and defined using ICD10-AM codes starting with “I50”, ICD-9 code starting with “428”, or SNOMED code mapped to an “I50” ICD10-AM code). SNOMED codes were mapped to ICD-10-AM codes using CSIRO’s SnoMAP Starter Web Service (https://ontoserver.csiro.au/site/our-solutions/snomap-starter/). Comorbidities were identified by their corresponding I-codes as in previous community-based analyses of heart failure-related comorbidities [16]. U-codes corresponding to respective comorbidities were included to ensure complete data capture, due to inconsistencies in coding of chronic diseases in Australian hospital data.

The primary outcome of the study was heart failure-related decompensation within 12-months, and defined as any heart failure-related re-presentation to ED, readmission to hospital, or all-cause mortality within 12- months of index HF-event.

#### Analysis

Statistical analysis was conducted utilising R statistical software (V3.6.1). Initial visual data interrogation and descriptive analyses were undertaken to determine the volume and pattern of ED presentations, hospital admissions, outpatient care, and mortality DP compared to PP.

Mean and standard deviation (SD) for normally distributed variables, and median and interquartile ranges (IQR) calculated for non-normally distributed variables. Differences between health service use by socio-demographic, clinical and health service characteristics were also determined. Socio-demographic characteristics included socioeconomic status (measured according to the index of relative socio-economic disadvantage [IRSD] where lower scores indicate greater disadvantage), remoteness (according to Australian Bureau of Statistics Geographical Area of Remoteness) [17], health insurance, sex, and age. Clinical and health service factors assessed for hospital admissions were length of stay, admission to intensive care unit (ICU) and length of ICU stay. For ED, these included mode of arrival (private or ambulance), triage acuity (dichotomised into “high acuity” [categories 1-3] and “low acuity” [categories 4-5], time spent in ED, whether seen “on time” according to national standards for each triage category [18], mode of separation (discharge versus admission); and for out-patients waiting time from referral to appointment, appointment time and contact mode (in-person or telehealth). In-hospital and community all-cause mortality was measured by linking admissions data to the RBDM.

Differences were determined using a t-test for continuous variables and chi- squared test for proportional variables. Significance was set at 0.05 and all tests were 2-tailed.

A Cox proportional hazard model was applied to determine the impact of the pandemic on time to heart failure-related adverse events (heart failure-related ED presentation, hospital admission, or all-cause mortality). Models were presented unadjusted and adjusted for age, sex, socio-economic status, remoteness, and number of comorbidities. Further modelling was undertaken to determine whether socio-demographic characteristics impacted time to heart failure decompensation. Models included patients with complete data available for all variables analysed. No imputation of missing data was undertaken.

## Results

### Hospital admissions and ED presentations

Overall, 24,072 patients were admitted to NSW hospitals with a primary diagnosis of heart failure DP and 23,145 PP. Admission volumes were similar across age, sex, socio-economic and rurality strata (Table 1). In-hospital mortality increased by 0.3% (PP = 1,622/24,072 [6.7%], DP = 1,619/23,145 [7.0%], *p* < 0.01). Length of stay, length of ICU stay and likelihood of ICU admission were stable (Table 1).

**Table 1:** Demographic characteristics of patients admitted and presenting to emergency with heart failure pre COVID-19 compared to during COVID-19 in NSW, Australia

**Characteristic**	**Hospitalisation** **PP** *N* = 24,072	**Hospitalisation** **DP** *N* = 23,145	***p*-value**	**Emergency** **PP** *N* = 15,324	**Emergency** **DP** *N* = 15,023	***p*-value**
Age (median, IQR)	81.9 (73.3, 88.3)	82.2 (73.9, 88.2)	0.069	81.1 (72.2, 87.6)	81.1 (72.7, 87.5)	0.3
Sex			0.7			0.11
Female	11,173 (46.4%)	10,783 (46.6%)		7,167 (46.8%)	6,890 (45.9%)	
Male	12,899 (53.6%)	12,362 (53.4%)		8,157 (53.2%)	8,133 (54.1%)	
Remoteness Area			0.082			0.005
Major City	17,236 (71.8%)	16,398 (70.9%)		10,253 (67.1%)	9,835 (65.6%)	
Inner Regional	5,045 (21.0%)	4,942 (21.4%)		3,781 (24.7%)	3,958 (26.4%)	
Remote or very remote	1,739 (7.2%)	1,776 (7.7%)		1,251 (8.2%)	1,210 (8.1%)	
IRSAD (decile)			< 0.01			0.13
More disadvantaged (1-4)	11,284 (47.0%)	11,324 (49.0%)		8,447 (55.3%)	8,456 (56.4%)	
More advantaged (7-10)	8,903 (37.1%)	8,937 (38.6%)		3,559 (23.3%)	3,370 (22.5%)	
Private health insurance	9,302 (38.6%)		> 0.9	-	-	
Length of stay (hours)	203.4 (341.1)	200.2 (289.9)	0.3	-	-	
Attended ICU	1,142 (4.7%)	1,102 (4.8%)	> 0.9	-	-	
ICU length of stay (hours)	4.5 (51.4)	4.2 (37.8)	0.5	-	-	
In hospital mortality	1,622 (6.7%)	1,619 (7.0%)	< 0.001	-	-	
Mode of arrival						< 0.01
Private (car, foot)	-	-		6,625 (43.3%)	6,091 (40.6%)	
Public (ambulance)	-	-		8,547 (55.8%)	8,788 (58.5%)	
Triage acuity						0.3
High acuity^a^	-	-		4,824 (31.5%)	4,816 (32.1%)	
Low acuity^a^	-	-		10,500 (68.5%)	10,207 (67.9%)	
FCC within threshold						0.003
Timely	-	-		6,219 (40.6%)	6,350 (42.3%)	
Late	-	-		9,105 (59.4%)	8,673 (57.7%)	
Wait until finished (mins)	-	-		236.0 (178.0, 345.0)	260.0 (186.0, 374.0)	< 0.001
Mode of separation						< 0.01
Discharged	-	-		2,538 (16.6%)	2,789 (18.6%)	
Admitted	-	-		12,783 (83.4%)	12,230 (81.4%)	

*PP* pre pandemic, *DP* during pandemic, *FCC* first clinical contact

^a^high acuity (triage category 1-3), low acuity (triage 4 or 5)

**Fig. 1:**
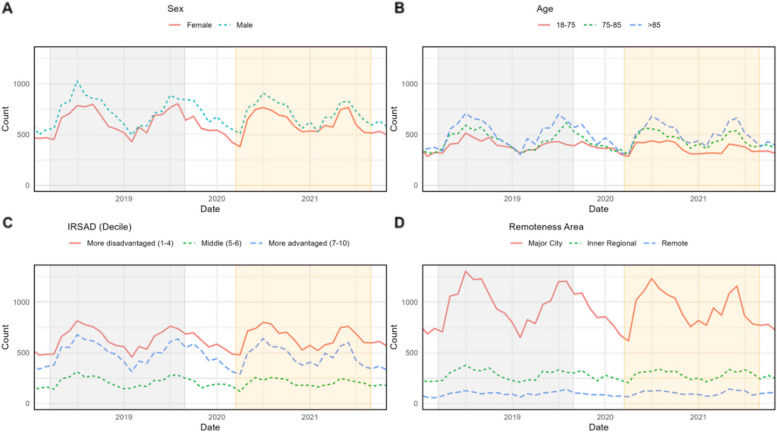
Number of patients admitted per week to NSW hospitals with a primary diagnosis of heart failure pre versus post pandemic stratified by sex (**A**), age (**B**), index of relative socio-economic disadvantage (IRSAD, **C**), Remoteness (**D**). Pre-pandemic – gray, pandemic – yellow

The volume of heart failure admissions was similar prior to and during the pandemic, however the pattern of seasonal variation differed with less variation observed among those aged 18-75 years compared to those in older age groups (Fig. 1B) and those residing in rural and remote areas versus major cities. Among those living in major cities, seasonal variation in hospitalisation was blunted DP versus PP (Fig. 1D).

**Fig. 2:**
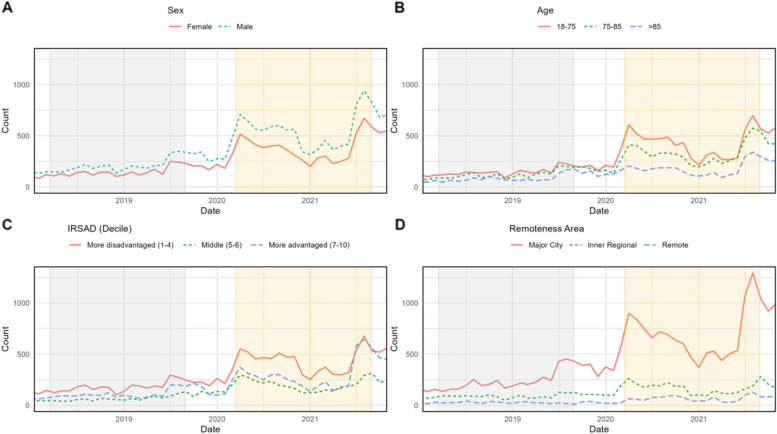
Volume of telehealth appointments among patients with a primary diagnosis of heart failure in NSW pre versus during pandemic stratified by sex (**A**), age (**B**), index of relative socio-economic disadvantage (IRSAD, **C**) and remoteness (**D**). Pre-pandemic = gray, pandemic - yellow

ED demographics were also similar between the periods (Table 1). A lower proportion of the states’ ED presentations occurred in major cities during the pandemic (PP = 10,253/15,324 [67.1%], DP = 9,835/15,023 [65.6%], *p* = 0.005) and more arrivals were via ambulance (PP = 8,547/15,324 [55.8%], DP = 8,788/15,023 [58.5%], *p* < 0.001). Those presenting DP were more likely to be seen in a timely manner (PP = 6,219/15,324 [40.6%], DP = 6,350/15,023 [42.3%], *p* = 0.03) despite similar level of acuity (see Table 2). A lower proportion of ED presentations for heart failure were admitted during the pandemic (PP = 12,783/15,324 [83.4%], DP = 12,230/15,023 [81.4%], *p* < 0.01) Fig. 2.

**Table 2:** Comorbidities of patients admitted to hospital with heart failure in NSW, Australia pre-pandemic compared to during pandemic ^a^n (%); Median (IQR)

**Characteristic**	**Before pandemic** *N* = 13,337^a^	**During pandemic** *N* = 13,000^a^	***p*-value**
Cancer	1,411 (10.6%)	1,533 (11.8%)	0.002
Diabetes mellitus Type 2	5,228 (39.2%)	5,028 (38.7%)	0.4
Obesity	3,329 (25.0%)	3,624 (27.9%)	< 0.001
Depression/Anxiety	2,776 (20.8%)	2,901 (22.3%)	0.003
Sleep disorders	1,142 (8.6%)	1,143 (8.8%)	0.5
Primary hypertension	11,370 (85.3%)	11,269 (86.7%)	< 0.001
Myocardial infarction	1,570 (11.8%)	1,525 (11.7%)	> 0.9
Atrial fibrillation and flutter	6,151 (46.1%)	5,799 (44.6%)	0.014
Ischemic stroke	380 (2.8%)	338 (2.6%)	0.2
Chronic bronchitis and COPD	3,765 (28.2%)	3,871 (29.8%)	0.006
Asthma	1,978 (14.8%)	2,192 (16.9%)	< 0.001
Chronic kidney disease	4,891 (36.7%)	4,747 (36.5%)	0.8
Count of comorbidities	27.0 (16.0, 43.0)	30.0 (18.0, 47.0)	< 0.001
Count of comorbidities			< 0.001
1-4	288 (2.2%)	180 (1.4%)	
5-8	862 (6.5%)	682 (5.2%)	
9-13	1,479 (11.1%)	1,224 (9.4%)	
> 13	10,708 (80.3%)	10,914 (84.0%)

The median number of comorbidities among patients admitted with heart failure was higher DP compared to PP (median [IQR] DP = 30.0 [18.0, 47.0], PP = 27.0 [16.0, 43.0] *p* < 0.001. A higher proportion of patients admitted with cancer, obesity, depression/anxiety, hypertension, chronic obstructive pulmonary disease, and asthma DP (Table 2). Fewer patients admitted had comorbid AF DP (PP = 5,770/13,000 [44.6%], DP = 6,151/13,337 [46.1%], p 0.014).

### Non-admitted care

There were fewer non-admitted care services recorded DP compared to the pre-pandemic period (PP = 44,447, DP = 35,801 occasions of service), but the proportion of services provided via telehealth increased nearly three-fold (PP = 5,978/44,447 [13.4%], DP = 15,901/35,081 [44.4%], Table 3). Telehealth uptake was greater among males, those living in more advantaged areas, and urban populations (Table 3). Demographics of patients during and pre-pandemic were similar, but more outpatient services were provided to disadvantaged patients during the pandemic compared to pre-pandemic (PP = 18,751/44,447 [42.2%], DP = 16,454/35,801 [46.0%], *p* < 0.001). Waiting times and appointment lengths were shorter during the pandemic.

**Table 3:** Non-admitted service provision in NSW pre compared to during COVID-19

**Characteristic**	**Before pandemic**, *N* = 44,447	**During pandemic**, *N* = 35,801	***p*-value**
Age (median, IQR)	76 (68, 83)	76 (67, 83)	0.01
Sex			< 0.01
Female	17,921 (40.3%)	13,952 (39.0%)	
Male	26,526 (59.7%)	21,849 (61.0%)	
Remoteness Area			< 0.01
Major City	31,749 (71.4%)	25,214 (70.4%)	
Inner Regional	9,463 (21.3%)	7,386 (20.6%)	
Rural, remote or very remote	3,235 (7.3%)	3,201 (8.9%)	
IRSD (decile)			< 0.01
More disadvantaged (1-4)	18,751 (42.2%)	16,454 (46.0%)	
Middle (5-6)	7,575 (17.0%)	7,239 (20.2%)	
More advantaged (7-10)	18,121 (40.8%)	12,108 (33.8%)	
Wait time (days)	50 (9, 125)	45 (8, 144)	0.6
Service length (hours)	1.00 (0.50, 1.50)	0.50 (0.25, 1.00)	< 0.001
Contact mode			< 0.001
In person	37,147 (83.6%)	18,339 (51.2%)	
Telehealth	5,978 (13.4%)	15,901 (44.4%)	
Services per person	3 (1, 6)	3 (1, 6)	

*IRSAD* index of relative socio-economic disadvantage

### Readmission, representation, and mortality

Risk of heart failure-related adverse events (any of heart failure-related ED presentation, heart failure-related hospitalisation, or all-cause mortality) following an index heart failure-related hospital admission was lower DP compared to PP (Adjusted HR = 0.91 [95% CI 0.88, 0.95]; Table 4). There was no significant interaction between sex, age, socio-economic status or rurality and the pandemic period. Time to first heart failure-related adverse event was shorter PP than DP (median [IQR] PP = 273 [259, 290] days, DP = 323 [300, 342] days, Table 4).

**Table 4:** Factors associated with heart failure related adverse events (heart failure-related ED representation, hospital admission, or all-cause mortality)

	**Unadjusted**			**Adjusted**^**b**^		
**Characteristic**	**HR**^a^	**95% CI**^a^	***p*-value**	**HR**^a^	**95% CI**^a^	***p*-value**
Timepoint						
Before Pandemic	1.00	—		1.00	—	
During Pandemic	0.92	0.88, 0.95	< 0.001	0.89	0.86, 0.93	< 0.001
Sex						
Female				1.00	—	
Male				1.12	1.08, 1.17	< 0.001
Age bracket						
18-75				1.00	—	
75-85				1.24	1.18, 1.31	< 0.001
> 85				1.59	1.51, 1.68	< 0.001
IRSAD^c^ (decile)						
More disadvantaged (1-4)				1.00	—	
Middle (5-6)				0.95	0.90, 1.01	0.10
More advantaged (7-10)				0.91	0.87, 0.95	< 0.001
Remoteness Area						
Major City				1.00	—	
Inner Regional				0.95	0.90, 1.00	0.033
Remote				0.97	0.89, 1.05	0.4
Comorbidities				1.01	1.00, 1.01	< 0.001

^a^*HR* Hazard Ratio, *CI* Confidence Interval

^b^Adjusted for sex, age, socio-economic status, remoteness and number of comorbidities

^c^Index of relative socioeconomic advantage and disadvantage

Following an index hospital admission, women had a longer time to heart failure related representation, readmission, or all-cause mortality than men across the period of observation (median [IQR] 313 [293, 335] vs 281 [265, 297] days). Time to event was shorter with advancing age, however socio-economic status and rurality were not predictive of time to event.

## Discussion

The current analyses compared healthcare utilisation trends among patients with heart failure during and before the pandemic and found volume and demographics of patients admitted to hospital and presenting to ED were largely unchanged during the pandemic in Australia. This finding sits in contrast to several studies that describe reduced volume of heart failure admissions internationally [8, 19, 20], and is likely a reflection of reduced COVID-19 burden translating to reduced hesitancy to seek care in Australia. Time to heart failure-related decompensation was slightly increased DP, suggesting adequate post discharge care without significantly increased out of hospital mortality. There was a slightly increased proportion of patients arriving by ambulance and time to clinical contact or medical assessment, an important measure of ED care quality in Australia [18], was marginally shorter DP. Overall time in ED was increased by approximately 10%. Acuity of presentation and proportion admitted to intensive care was unchanged, but those admitted with heart failure during the pandemic were older and suffered from a slightly higher burden of comorbidities, which may explain the small increase in DP in-hospital mortality.

Telehealth was a largely new health service offering during the pandemic in Australia and made widely available in both high and low case-load settings. The current analyses describe a small reduction in non-admitted outpatient care during the pandemic, primarily among socio-economically advantaged patients. This could be due to access to alternative and privately funded care among wealthier patients, which was not captured in this analysis. Paradoxically, uptake of publicly funded telehealth services was highest among the most socio-economically advantaged and those in major cities. This suggests a relative gap in the provision of telehealth-enabled care for socio-demographically vulnerable patients during the pandemic, which has also been described elsewhere [21]. Telehealth services may be less accessible to the most vulnerable for various reasons including the need for home technology and supportive home environments. Several pre-pandemic analyses highlight the cost saving potential of telehealth-enabled models of heart failure care [22, 23], but there are few examples of contemporarily studies and most studies have occurred in high income countries.

As is now recognised the influence of COVID-19 extended far beyond counts of case numbers, vaccination rates, and mortality [24]. Policies focused on curbing the spread of disease had widespread social, economic, and environmental impact [25]. Patients with heart failure are older, less socio-economically advantaged, and saddled with a high burden of comorbid disease [26]. We hence hypothesized they would be more vulnerable to changes in health policy. This study provides a unique lens into the impact of drastic system-level changes implemented during the pandemic on a vulnerable cardiovascular population without the cofounder of significant COVID-19-related morbidity and mortality.

Seasonal variation in heart failure and coronary heart disease presentations is a well-described phenomenon, with peaks observed during winter and troughs in summer. Early literature suggested this was due to increased winter viruses precipitating exacerbations [27], however several contemporary analyses demonstrate the changes occur independent of viral load and may be due to physiologic adaptations in low ambient temperature, increased sodium intake during winter months, or another mechanism yet undescribed [28, 29]. This analysis found seasonal variation persisted during the pandemic, but was less pronounced among younger patients and those residing in rural or remote regions. This may be due to milder winter seasons in rural and remote areas of NSW or the greater physiologic reserve of young patients with heart failure.

Acuity on presentation to ED was unchanged with a similar proportion of patients across all urgency statuses. This sits in contrast to a NSW-wide analysis published early in the pandemic that found a 34% increase in lowest-acuity presentations (triage category 5) with concomitant decreases in other triage categories [30]. This difference in findings may be due to more homogenous acuity mix among patients with heart failure requiring ED care.

A study of ED presentations during the pandemic in a tertiary hospital in Australia and Korea found stable total length of stay despite decreased numbers of ED presentations, suggesting changes to ED process during the pandemic increased total time in ED [31]. Time to representation, readmission or all-cause mortality was longer during the pandemic with no significant interaction among socio-demographic subgroups of interest, possibly suggestive of better heart failure control during the pandemic in spite of reduced volumes of in-hospital care.

### Limitations

The current study presents analysis of a large cohort of all heart failure related presentations to public and private hospitals in NSW, the most populous state in Australia, and has several strengths and limitations. The health services data while comprehensive in coverage, was limited by what is recorded in the electronic medical record and thus lacked important clinical details on disease severity and precipitant of presentation. Limited data on comorbidities is presented with exploratory analysis, but was not the focus of this study and is not incorporated as covariates in primary outcome models. Lockdown periods and COVID-19 caseloads were considered in the analysis, but direct linkage to COVID-19 databases to control for cofounding from concomittant infection was not possible. Non-admitted patient data only captured non-admitted care delivered in public hospitals and therefore comment on the impacts of or changes to delivery of care in the private system, which comprises a signifciant minority of healthcare delivery in Australia, was not possible.

## Conclusion

The COVID-19 pandemic impacted heart failure patient interaction with healthcare services consequent to both changes in policy and COVID-19 pandemic load. However, overall volume and demographic mix of presentations was similar to the pre-pandemic era. Small increases in total ED time did not occur in conjunction with increased presentation acuity, despite a higher proportion of attendances via ambulance. Rapid uptake of telehealth enabled provision of care that adhered to restrictions, however disparities in service uptake may have exacerbated existing inequity for those living in rural, remote, and areas of higher socio-economic disadvantage.

## Data Availability

Data are and will be made available after review of a reasonable request to be submitted to the corresponding author at daniel.mcintyre@sydney.edu.au.

## Abbreviations

APDC: Admitted Patient Data Collection
CHeReL: Centre for Health Record Linkage
CODURF: Cause of Death Unit Record File
DP: During pandemic
ED: Emergency Department
EDDC: Emergency Department Data Collection
NSW: New South Wales
PP: Pre pandemic
ICD: International classification of diseases
ICU: Intensive care unit
IQR: Interquartile range
IRSD: Index of relative socio-economic disadvantage
NAP: Non-Admitted Patients data collection
RBDM: Registry of births, deaths, and marriages
SD: Standard deviation
